# Cepharanthine as an effective small cell lung cancer inhibitor: integrated insights from network pharmacology, RNA sequencing, and experimental validation

**DOI:** 10.3389/fphar.2024.1517386

**Published:** 2024-11-28

**Authors:** Fengyun Zhao, Zhaowei Ding, Mengting Chen, Mingfang Ji, Fugui Li

**Affiliations:** ^1^ Cancer Research Institute of Zhongshan City, Zhongshan City People’s Hospital, Zhongshan, Guangdong, China; ^2^ Department of Hepatobiliary Surgery, The First Affiliated Hospital of Guangzhou Medical University, Guangzhou, Guangdong, China; ^3^ South China Normal University, Guangzhou, Guangdong, China

**Keywords:** small cell lung cancer, cepharanthine, network pharmacology, cholesterol metabolism, RNA sequencing

## Abstract

**Background:**

Small cell lung cancer (SCLC) is an aggressive malignancy with limited treatment options and poor prognosis, underscoring the need for new therapeutic agents.

**Methods:**

A library of 640 natural products was screened for anti-proliferative activity in SCLC cells. The effects of Cepharanthine (CE) on SCLC cells were assessed *in vitro* and *in vivo*. Network pharmacology and RNA sequencing (RNA-seq) were used to elucidate the molecular mechanisms. Pathway enrichment analysis was performed using Gene Set Enrichment Analysis (GSEA) with Hallmark and Reactome gene sets. Protein-protein interaction (PPI) networks, along with the Cytoscape cytoHubba plugin, were used to identify key hub genes. RT-PCR and Western blotting were employed to validate mRNA and protein expression. Molecular docking studies assessed the binding affinity of CE to potential targets. Bioinformatics analyses, including expression profiling, prognostic evaluation, and loss-of-function studies, were used to explore the role of specific genes in SCLC.

**Results:**

CE was identified as a promising SCLC inhibitor. *In vitro*, CE significantly inhibited SCLC cell proliferation, colony formation, migration, and invasion, while promoting apoptosis. *In vivo*, CE treatment notably reduced tumor volume in xenograft models. Network pharmacology identified 60 potential target genes, with enrichment analysis indicating their involvement in cholesterol metabolism regulation. RNA-seq and experimental validation further confirmed that CE inhibits cholesterol synthesis in SCLC cells by downregulating key enzymes, including HMGCR, HMGCS1, IDI1, FDFT1, and SQLE. Molecular docking studies confirmed the binding of CE to these enzymes. Additionally, these enzymes were found to be highly expressed in SCLC cells, with elevated levels of HMGCS1, HMGCR, and IDI1 correlating with poor prognosis. Functional assays revealed that silencing these genes significantly suppressed SCLC cell proliferation.

**Conclusion:**

This study identifies CE as a potential therapeutic agent for SCLC, acting through the suppression of cholesterol synthesis, and uncovers novel therapeutic targets for the treatment of this aggressive cancer.

## 1 Introduction

Small cell lung cancer (SCLC) is a highly aggressive subtype of lung cancer, accounting for approximately 15% of all cases (Yin et al., 2022). Characterized by rapid growth, early metastasis, and frequent recurrence, SCLC is associated with a poor prognosis (Wang et al., 2023). Despite the availability of standard treatment options such as chemotherapy and radiation therapy, the overall survival rate remains low, with a median survival of less than 1 year for most patients (Herzog et al., 2021). The limited effectiveness of current therapies underscores the urgent need for novel treatment strategies to improve patient outcomes.

Natural compounds have emerged as promising candidates for developing novel anti-cancer agents. These compounds, derived from various biological sources, present several advantages, including broad availability, ease of extraction, and generally lower toxicity compared to synthetic drugs (Scaria et al., 2020). Numerous studies have reported their potential in inhibiting tumor growth, promoting apoptosis, and enhancing the efficacy of existing therapies (Naeem et al., 2022). Recent studies have highlighted the therapeutic potential of several natural compounds in SCLC. For instance, oridonin induces autophagy via the PERK/eIF2α/CHOP signaling pathway, suggesting enhanced therapeutic responses when combined with a PERK inhibitor (Xu et al., 2024). Screening of a natural product library identified tubercidin and lycorine HCl as promising anti-SCLC agents (Chen et al., 2022). Additionally, the composite formulation Ocoxin^®^ oral solution (OOS) displayed dose-dependent anti-SCLC effects *in vitro* and *in vivo*, primarily through inhibiting cell proliferation and inducing cell death via caspase activation. OOS also enhances the efficacy of standard SCLC therapies, highlighting its potential as a complementary treatment strategy (Diaz-Rodriguez et al., 2018). Collectively, these studies underscore the promise of natural compounds in developing more effective treatments for SCLC. In this study, we screened a library of 640 natural compounds and identified Cepharanthine (CE) as a promising candidate with significant inhibitory effects on SCLC cell proliferation.

Previous research has demonstrated that Cepharanthine (CE) possesses anti-inflammatory, antiviral, and anti-cancer activities in various cancers (Liu et al., 2023a). It inhibits hepatocellular carcinoma growth by modulating amino acid metabolism (Feng et al., 2021), regulates osteoclastogenesis via NF-κB and NFAT pathways (Lin et al., 2019), and alleviates titanium particle-induced osteolysis by modulating the OPG/RANKL ratio *in vivo* (Liao et al., 2019). As a regulator of the Keap1-Nrf2 pathway, CE inhibits gastric cancer growth through oxidative stress and energy metabolism (Lu et al., 2023a). Additionally, CE sensitizes triple-negative breast cancer cells to epirubicin by promoting cofilin oxidation and apoptosis (Shen et al., 2022), and induces ferroptosis in Non-small cell lung cancer (NSCLC) by inhibiting NRF2, leading to ER stress (Bai et al., 2024). Despite these diverse anti-cancer effects, its specific impact on SCLC remains largely unexplored.

Herein, we demonstrated that CE exhibits potent anti-SCLC effect. *In vitro*, CE inhibits SCLC cell proliferation, migration, and invasion, while promoting apoptosis in SCLC cells. *In vivo*, CE significantly reduces tumor volume in xenograft models. Network pharmacology and RNA-seq revealed that CE suppresses cholesterol synthesis in SCLC cells by downregulating key rate-limiting enzymes (HMGCS1, HMGCR, IDI1, FDFT1, and SQLE). It was further established that these enzymes are highly expressed in SCLC cell lines, with elevated levels of HMGCS1, HMGCR, and IDI1 correlating with poor prognosis in SCLC patients. Moreover, silencing these genes reduces SCLC cell proliferation. Our findings position CE as a novel therapeutic agent targeting cholesterol biosynthesis, offering potential new therapeutic targets for improving patient outcomes in this aggressive cancer.

## 2 Materials and methods

### 2.1 Acquisition of relevant targets of CE and SCLC-related genes

Structural information for CE was obtained from PubChem (https://pubchem.ncbi.nlm.nih.gov/). The potential targets of CE were obtained through Super-PRED (https://prediction.charite.de/index.php), Similarity ensemble approach (SEA, https://sea.bkslab.org/), ChEMBL (https://www.ebi.ac.uk/chembl/), PharmMapper (http://www.lilab-ecust.cn/pharmmapper/), and Swiss Target Prediction database (http://www.swisstargetprediction.ch/). After compiling the identified targets and removing duplicates, 674 potential targets of CE were established. The SCLC-related genes were identified by intersecting the genes associated with SCLC from the CTD (https://ctdbase.org/) and GeneCards databases (https://www.genecards.org/) with the differentially expressed genes (DEGs) obtained from the analysis of SCLC datasets (GSE149507, GSE40275, GSE60052) using the GEO2R tool of the Gene Expression Omnibus (GEO, https://www.ncbi.nlm.nih.gov/geo/query/acc.cgi). To determine the intersection of CE targets and SCLC-related genes, a venn diagram was generated using the online tool available at http://bioinformatics.psb.ugent.be/webtools/Venn/. This intersection highlights the potential targets for the anti-SCLC effects of CE.

### 2.2 Protein-protein interaction (PPI) network construction and analysis

Sixty potential targets of CE identified through network pharmacology were imported into the STRING database (http://string-db.org) to construct the PPI network. For this analysis, the following parameters were set: the organism was selected as *Homo sapiens*, which corresponds to human proteins; the minimum required interaction score was set to 0.7 to ensure the inclusion of interactions with high confidence; and the option to hide disconnected nodes in the network was enabled to focus on the most relevant protein interactions and reduce noise from isolated proteins. After constructing the initial PPI network, the network data was exported in tab-separated values (TSV) format for further analysis in Cytoscape (version 3.10.1, https://cytoscape.org/). Within Cytoscape, the CytoHubba plugin was utilized to identify and analyze the hub genes in the PPI network. CytoHubba is a powerful tool that uses various algorithms to determine the most important nodes (hub genes) based on their interaction patterns in the network. All parameters were set to their default values in CytoHubba to maintain consistency and avoid bias in the analysis.

### 2.3 Pathway enrichment analysis

Gene Set Enrichment Analysis (GSEA) was performed to identify enriched signaling pathways. Gene list was analyzed using Metascape (www.metascape.org/) for Gene Ontology Biological Process terms (GO BP). The resulting enrichment files were imported into the Cytoscape using the EnrichmentMap app for visualization. Parameters for visualization were set to default values, unless otherwise specified.

### 2.4 Cell culture and reagents

Human SCLC cell lines NCI-H1688, NCI-H146, and NCI-H446, as well as human lung bronchial epithelial cell line 16HBE and BEAS-2B, were obtained and authenticated from the Shanghai Institute of Cell Biology, Chinese Academy of Sciences (Shanghai, China). All cell lines were maintained in either RPMI-1640 medium or DMEM medium supplemented with 10% fetal bovine serum and 5% penicillin/streptomycin. Cells were cultured at 37°C in a humidified incubator with 5% CO_2_. The natural compound library and Cepharanthine were purchased from TargetMol (Shanghai, China). CE was stored at −20°C as a 10 mM stock solution in dimethyl sulfoxide (DMSO) and diluted in culture medium to achieve desired concentrations. The final DMSO concentration was maintained below 0.1%, which is non-toxic to cells.

### 2.5 Cell proliferation assay

Cell proliferation was determined by using Cell Counting Kit-8 (CCK-8) (Dojindo Molecular Technologies, Inc.). Cells were seeded at 3 × 10^3^ cells/well in 96-well plates. After 24 h, cells were treated with various concentrations of the compounds. Absorbance at 450 nm was measured at different time points to assess cell viability. Each condition was tested in triplicate. The data were normalized to the untreated control.

### 2.6 Colony formation assay

For the colony formation assay, 1 × 10^3^ cells were seeded in six-well plates. After 24 h, cells were treated without or with CE (1 µM). The medium was replaced every 2 days. After 14 days of incubation, colonies were fixed with 4% formaldehyde and stained with 0.1% crystal violet. Colonies greater than 0.1 mm in diameter were counted using an inverted microscope.

### 2.7 Cell apoptosis assay

Cells were plated in 6 cm dishes and treated without or with CE (1 µM) for 24 h. Apoptosis was assessed using the FITC Annexin-V Apoptosis Detection Kit (BD, catalog number 556547) according to the manufacturer’s protocol. Cells were analyzed by flow cytometry using the Accuri C6 flow cytometer (BD). Data analysis was performed with FlowJo software.

### 2.8 Transwell cell migration and invasion assay

For migration assays, 2 × 10⁴ cells were plated in the upper chamber of a 24-well Transwell plate (Corning, catalog number 3422) with an 8 μm pore size. For invasion assays, the upper membrane was coated with Matrigel (Corning, catalog number 354234). The lower chambers were filled with complete medium. Cells were incubated for 24 h. Afterward, cells were fixed with 4% formaldehyde, stained with 0.1% crystal violet and counted under light microscope. The data are presented as the average number of cells migrated or invaded from three independent experiments.

### 2.9 Western blot

Cells were lysed with RIPA buffer (Cell Signaling Technology, catalog number 9806), supplemented with protease inhibitor cocktail (Thermo Fisher Scientific, catalog number 78429). Protein concentrations were measured with a BCA Protein Assay Kit (Thermo Fisher, catalog number 23227). Equal amounts of protein (10 μg) were separated by 8%–12% SDS-PAGE and transferred to a PVDF membrane (Millipore).

Membranes were blocked with 5% non-fat dry milk in TBST for 1 h, followed by overnight incubation with primary antibodies: SQLE (A2428), FDFT1 (A4651), HMGCS1 (A3916), and HMGCR (A1633) (Abclonal Technology, Wuhan, China); IDI1 (ab97448) and GAPDH (ab9485) (Abcam, Cambridge, UK). The membranes were incubated with secondary antibodies (HRP-conjugated anti-rabbit or anti-mouse) and visualized using SuperSignal West Dura Chemiluminescent Substrate (Thermo Fisher Scientific, catalog number 37071).

### 2.10 Quantitative real-time PCR (qRT-PCR)

Total RNA was isolated using the Direct-zol RNA Kit (Zymo Research, catalog number R2050) according to the manufacturer’s protocol. cDNA was reverse-transcribed with the StarScript II First-strand cDNA Synthesis Kit (GenStar, catalog number A212-05). Real-time PCR (RT-PCR) was performed using the 2 × RealStar Green Fast Mixture (GenStar, catalog number A301-01), and amplification was performed in a 96-well format in triplicate using the CFX96 Real-Time PCR detection system (Bio-Rad). The relative mRNA expression of each gene was normalized to β-actin RNA levels and analyzed using the 2^-△△Ct^ method. The following primers were used to measure specific target genes. SQLE: 5′-CTC​CAA​GTT​CAG​GAA​AAG​CCT​GG-3′ and 5′- GAG​AAC​TGG​ACT​CGG​GTT​AGC​T-3′; FDFT1: 5′- TGT​GAC​CTC​TGA​ACA​GGA​GTG​G-3′ and 5′- GCC​CAT​AGA​GTT​GGC​ACG​TTC​T -3′; IDI1: 5′- GCC​GCA​GAC​TGT​GCT​CAA​AGC -3′ and 5′- CCT​GTT​GCT​TGT​CGA​GGT​GGT​T -3′; HMGCS1: 5′- AAG​TCA​CAC​AAG​ATG​CTA​CAC​CG-3′ and 5′- TCA​GCG​AAG​ACA​TCT​GGT​GCC​A-3′; HMGCR: 5′- GAC​GTG​AAC​CTA​TGC​TGG​TCA​G -3′ and 5′- GGT​ATC​TGT​TTC​AGC​CAC​TAA​GG -3′; β-actin: 5′-TCG​TGC​GTG​ACA​TTA​AGG​AG-3′ and 5′-ATG​CCA​GGG​TAC​ATG​GTG​GT-3′.

### 2.11 Xenograft model

The animal care and procedures in this study were approved by the Institutional Animal Care and Use Committee of Zhongshan City People’s Hospital (Approval number: K2023-007). Six-week-old BALB/C nude mice were used to investigate the *in vivo* anti-SCLC effect of CE. All mice were kept under specific pathogen free (SPF) conditions. 5 × 10^5^ H1688 cells were subcutaneously into the flank of each mouse. Tumor size was measured with calipers every 3 days after tumor cell implantation. Tumor volume was calculated using the formula: (width^2^ × length)/2. When the tumor volume reached approximately 30 mm³, mice were randomly assigned to either a control group or the Cepharanthine treatment group (10 mg/kg) (Feng et al., 2021; Lu et al., 2023b). Treatments were carried out via intraperitoneal injection with either solvent or CE every 2 days. After 3 weeks, the mice were sacrificed, and their blood and tumor tissues were collected.

### 2.12 Cholesterol content measurement

The Amplex Red Cholesterol and Cholesteryl Ester Assay Kit (Beyotime Biotechnology, Catalog number S0211S) was utilized to quantify cholesterol levels according to the manufacturer’s guidelines. In this assay, cholesteryl esters are hydrolyzed by cholesterol esterase, releasing free cholesterol and fatty acids. The free cholesterol is subsequently oxidized by cholesterol oxidase, generating hydrogen peroxide (H₂O₂) and cholestenone. The resulting H₂O₂ reacts with Amplex Red to produce resorufin, whose fluorescence intensity or absorbance is directly proportional to the cholesterol concentration. A standard curve derived from the provided cholesterol standard solution was used to calculation the concentration of cholesterol, cholesteryl ester, and total cholesterol content in the samples. The fluorescence intensity was measured at an excitation wavelength of 530 nm and an emission wavelength of 590 nm.

### 2.13 Molecular docking

The molecular structure of CE (ZINC30726863) was downloaded from the ZINC20 database (https://zinc20.docking.org/). The 3D structure of the target protein was downloaded from the PDB database (https://www.rcsb.org). Water molecules and original ligands were removed from the protein structure using PyMOL 2.0 (https://pymol.org/2/). Molecular docking simulations were performed using the Maestro software (Schrödinger, LLC, New York, NY), and the results were visualized using PyMOL 2.0. The docking procedure involved preparation of both the ligand and protein structures, followed by the docking process using the default parameters. The binding energies of the ligand-protein complexes were calculated, and the top-ranked binding modes were visualized for further analysis.

### 2.14 RNA-sequencing

NCI-H1688 cells were treated with CE at a concentration of 1 µM for 24 h. Total RNA was extracted using the Direct-zol RNA kit (Zymo Research, Catalog number R2050) according to the manufacturer’s guidelines. The purity and quantification of RNA were assessed using the NanoDrop 2000 spectrophotometer (Thermo Scientific, Waltham, MA United States). RNA sequencing was conducted by Novogene (Beijing, China) using the Illumina HiSeq 4,000 platform. Raw sequencing reads were subjected to quality control using FastQC (https://www.bioinformatics.babraham.ac.uk/projects/fastqc/). Low-quality reads were filtered out, and the remaining high-quality reads were aligned to the *Homo sapiens* reference genome GRCh38 using STAR aligner (https://github.com/alexdobin/STAR).

Differentially expressed genes (DEGs) were determined using DESeq2 (https://bioconductor.org/packages/release/bioc/html/DESeq2.html), with a cut-off of Log_2_|Fold change| ≥ 1 and *P* < 0.05. To control for false positives, *P*-values were adjusted for multiple comparisons using the Benjamini-Hochberg method, maintaining a false discovery rate (FDR) threshold of 0.05. All analyses were conducted in R (version 4.2.1, https://www.r-project.org/).

### 2.15 Plasmid construction and transfection

Short hairpin RNA (shRNA) sequences were inserted into psiF-copGFP vectors (System Biosciences, Mountain View, CA, United States). The following shRNA sequences were used: SQLE (5′-GCT​CAG​GCT​CTT​TAT​GAA​TTA-3′), FDFT1 (5′-ACT​TGC​TAC​AAG​TAT​CTC​AAT-3′), IDI1 (5′-GCC​AGT​GGT​GAA​ATT​AAG​ATA-3′), HMGCS1 (5′-CCT​GAT​ATG​CTA​TCT​GAA​TAT-3′), HMGCR (5′-CCT​GTA​TAT​TTA​CTT​CCA​GTT-3′). The sequence for the negative control (shCtl) was 5′-GCT​CAG​GCT​CTT​TAT​GAA​TTA-3′. To generate stable cell lines, lentiviral particles were produced by transfecting HEK-293T cells with second-generation packaging vectors (pMD2. G and psPAX2, System Biosciences) using Lipofectamine 2000 (Thermo Fisher Scientific, catalog number 11668500). Lentiviral particles were harvested 48 h post-transfection. SCLC cell lines were infected with lentivirus in the presence of 8 μg/mL polybrene (Sigma-Aldrich,catalog number TR-1003) and selected with puromycin (2 μg/mL) for 7 days. The efficiency of transfection and stable expression was confirmed by GFP fluorescence under a fluorescence microscope.

### 2.16 Statistical analysis

All experiments were performed in triplicate, and data are presented as mean ± standard deviation (SD) unless otherwise stated. The distribution of data normality was evaluated using the D’Agostino-Pearson omnibus test. For comparisons between two groups, unpaired two-tailed Student’s t-tests were used for normally distributed data, while Mann-Whitney U tests were applied for data that did not follow a normal distribution. When comparing more than two groups, one-way analysis of variance (ANOVA) with Tukey’s multiple comparisons was utilized for normally distributed data, and the Kruskal-Wallis test with Dunn’s multiple comparisons test was employed for non-normally distributed data. In these cases, multiple comparisons were controlled using Tukey’s or Dunn’s tests, respectively, to reduce the likelihood of false positives. All statistical analyses were performed using appropriate tests based on the distribution of the data, and *p*-values <0.05 were considered statistically significant. The statistical significance was set at *P < 0.05, **P < 0.01, ***P < 0.001.

## 3 Results

### 3.1 Screening of the natural compound library revealed that cepharanthine possesses inhibitory effects against SCLC

**FIGURE 1:**
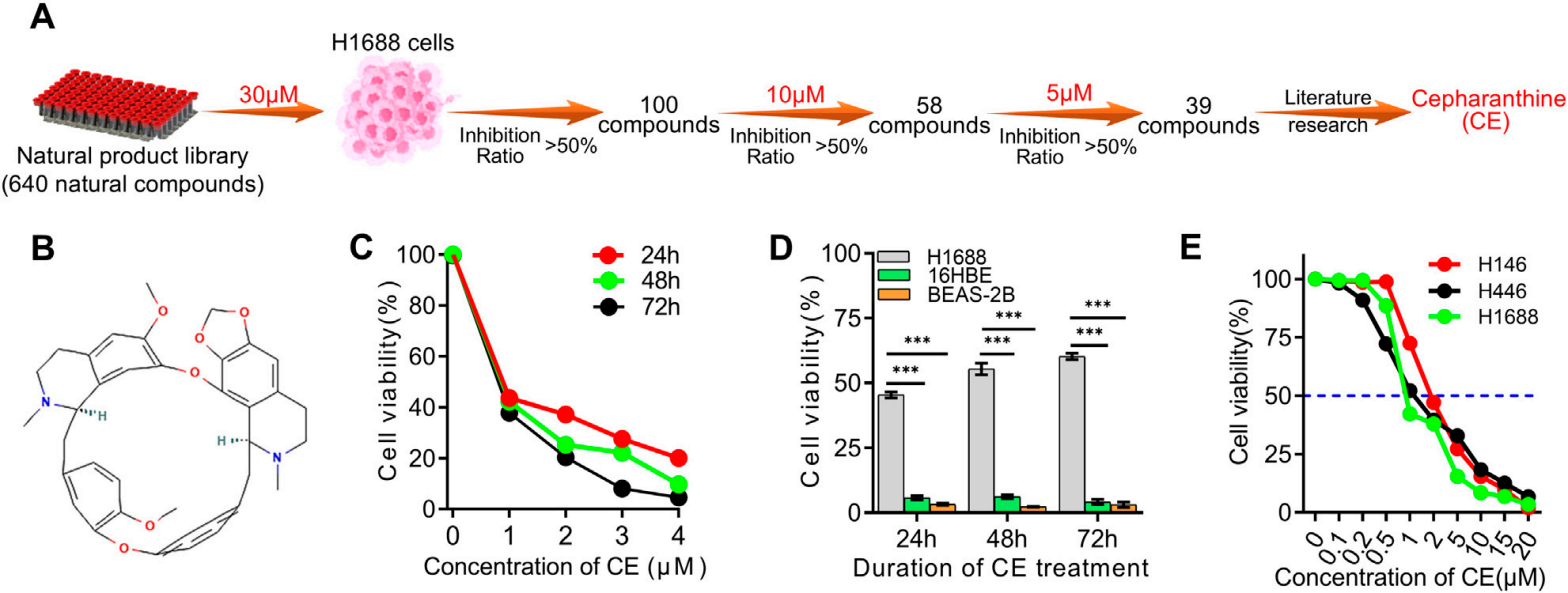
Screening of the natural compounds library revealed that Cepharanthine possesses inhibitory effects against SCLC. **(A)** Process of screening SCLC inhibitors using natural compounds library. **(B)** The chemical structure of Cepharanthine (CE, CAS: 481–49–2, Molecular Formula: C37H38N2O6; Formula Weight: 606.7 g/mol). **(C)** The inhibitory effects of CE on the proliferation of H1688 cells were evaluated. **(D)** The inhibitory effects of CE (1 µM) on the cell proliferation of H1688, 16HBE, and BEAS-2B cells after 24 h, 48 h and 72 h were assessed. 16HBE, human bronchial epithelial cell. BEAS-2B, human lung epithelial cell. **(E)** Cell proliferation was measured in SCLC cells after 24 h of treatment with different concentrations of CE, and the IC50 of CE in different SCLC cell lines was determined. Data are presented as means ± SD of three simultaneously performed experiments **(C–E)**. *P < 0.05, **P < 0.01, ***P < 0.001.

To identify novel compounds with activity against small cell lung cancer (SCLC), a library of 640 natural products was screened for their ability to inhibit SCLC cell proliferation (Supplementary Table S1; Figure 1A). Initially, H1688 SCLC cells were exposed to each compound at a concentration of 30 µM for 24 h, after which the inhibition ratio was evaluated using the Cell Counting Kit-8 (CCK-8) assay. Compounds demonstrating over 50% reduction in cell viability compared to the control group were selected for further analysis, resulting in the identification of 100 candidate compounds (Supplementary Table S2). These compounds were then tested at a concentration of 10 µM on H1688 cells, and the same assay was performed, resulting in the identification of 58 compounds for further evaluation (Supplementary Table S3). These 58 candidates were subsequently re-evaluated at a lower concentration of 5 μM, identifying 39 natural compounds that effectively inhibited H1688 cell proliferation (Supplementary Table S4). Upon literature review of these 39 compounds, Cepharanthine (CE) was identified as a compound of particular interest (Figure 1B). Although CE is known for its diverse medicinal properties, including antitumor activity, its role in SCLC has not been documented. Consequently, CE was selected for further investigation. The subsequent analysis focused on the effects of lower concentrations of CE on SCLC cell proliferation. At 1 μM, CE significantly inhibited the proliferation of H1688 cells (Figure 1C), while minimal effects were observed in normal human tissue cell lines (16HBE and BEAS-2B) (Figure 1D). The half-maximal inhibitory concentration (IC50) of CE in common SCLC cell lines was determined to be 0.8 µM for H1688, 1.1 µM for H446, and 1.5 µM for H146 cells (Figure 1E). These findings suggest that CE represents a promising candidate compound for the treatment of SCLC.

### 3.2 Cepharanthine inhibited SCLC cells *in vitro*

**FIGURE 2:**
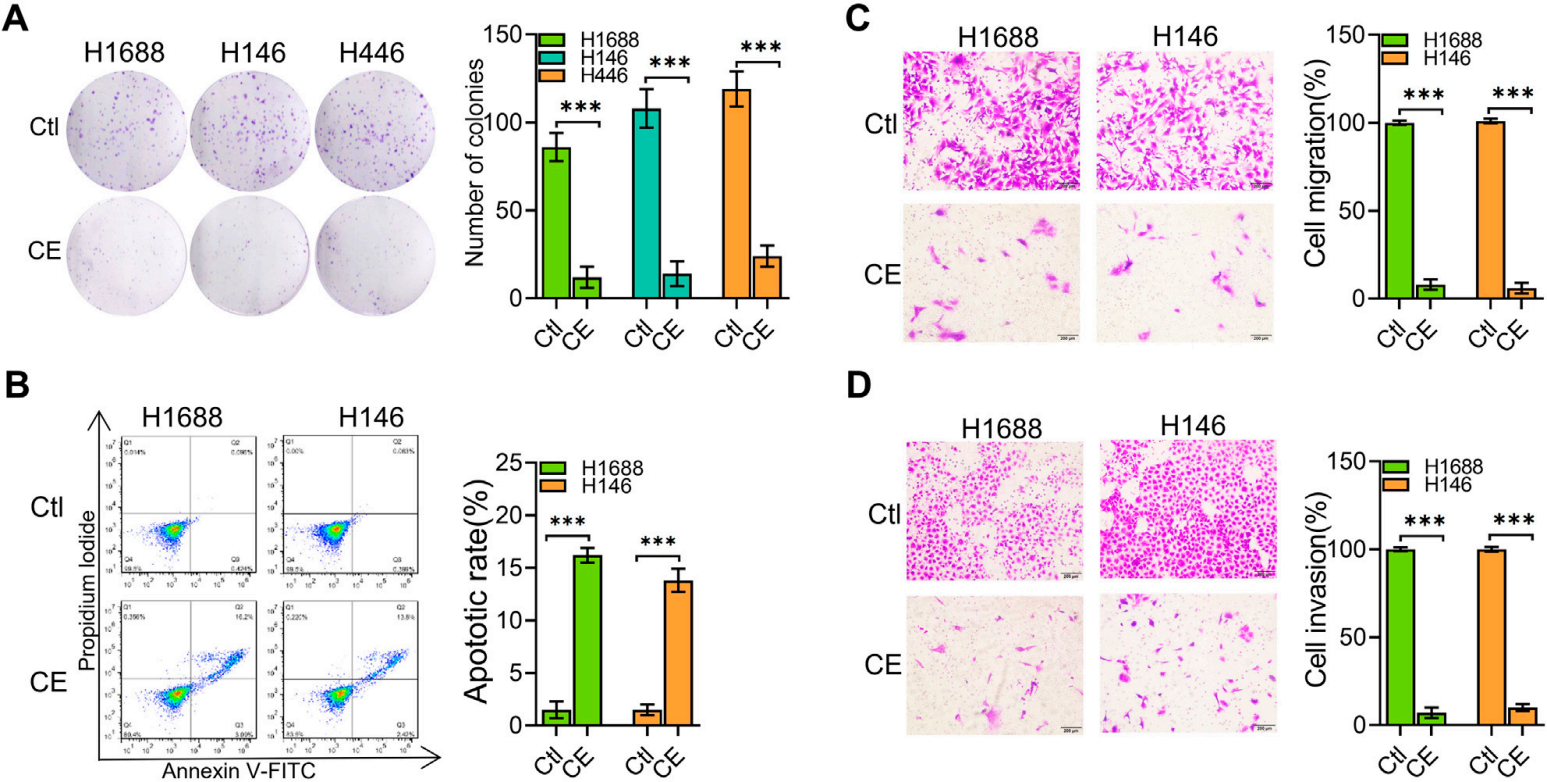
CE inhibited SCLC cells *in vitro*. **(A)** The colony formation assay was conducted to determine the inhibitory effect of CE on SCLC cells. The number of colonies was counted under a microscope. **(B)** SCLC cells were treated with Ctl (DMSO) or CE (1 µM) for 24 h, followed by Annexin V-FITC and PI staining and flow cytometry analysis. Both Annexin V-FITC and PI double-positive cells represent apoptotic cells. **(C, D)** The effect of CE (1 µM) on transwell migration **(C)** and invasion **(D)** of SCLC cells was assessed. Data are presented as mean ± SD of three simultaneously performed experiments **(A–D)**. *P < 0.05, **P < 0.01, ***P < 0.001.

To further investigate the anti-SCLC effects of CE, we explored its impact on several key aspects of SCLC cell behavior. Initially, the effect of CE on SCLC cell colony formation was analyzed, demonstrating significant inhibition of colony formation (Figure 2A). To determine how CE induces cell death in SCLC cells, the number of apoptotic cells was assessed using flow cytometry. The results indicated a significant increase in apoptotic cells in the CE-treated group (Figure 2B). Additionally, CE effectively suppressed the migration and invasion capabilities of SCLC cells (Figures 2C,D). Collectively, these results suggest that CE inhibits SCLC development *in vitro*, making it an ideal candidate for further mechanistic studies.

### 3.3 Network pharmacology analysis of the potential mechanism of CE against SCLC

**FIGURE 3:**
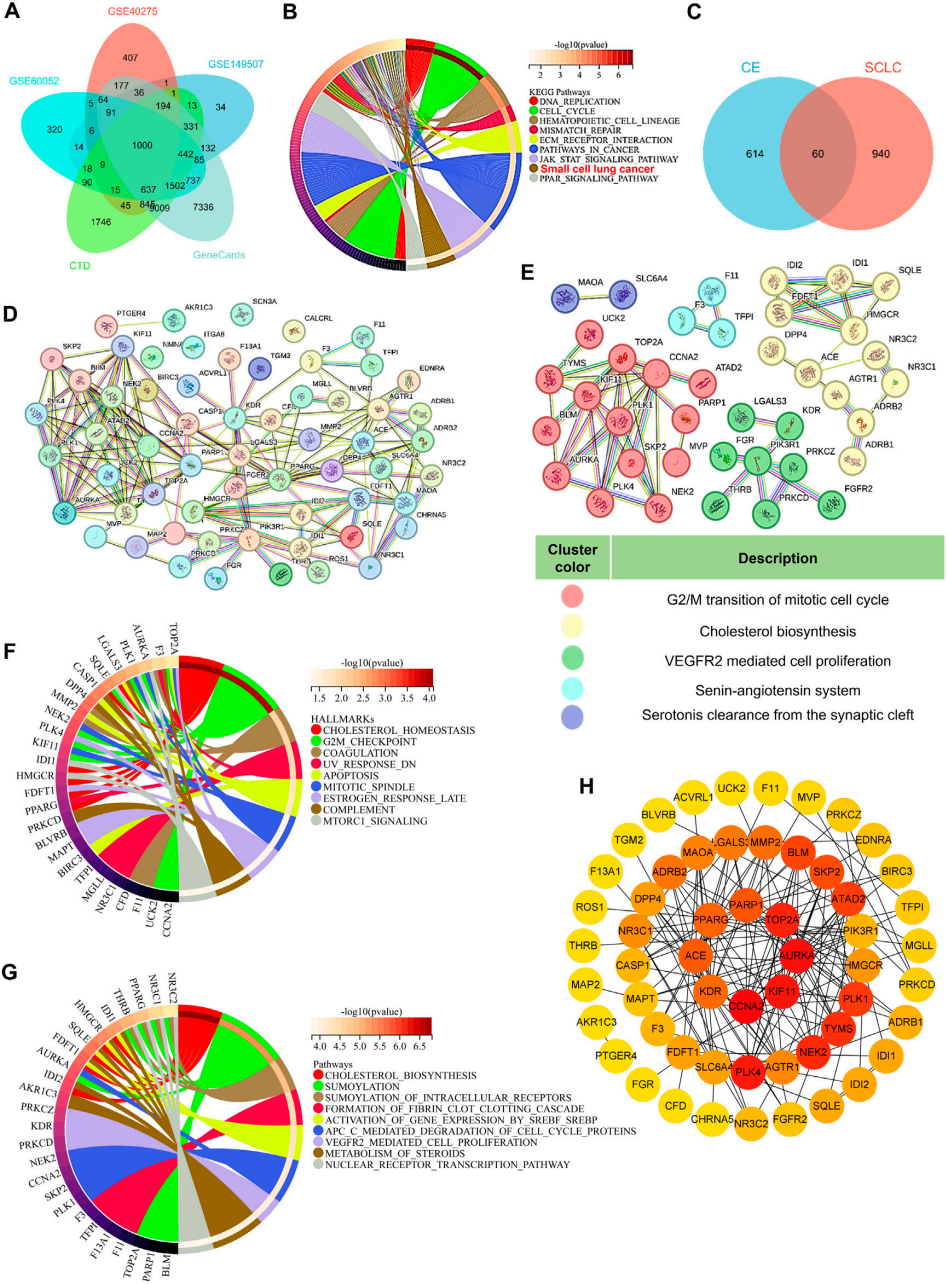
Network pharmacology analysis of the potential mechanism of CE against SCLC. **(A)** A Venn diagram was used to obtain SCLC-related genes. **(B)** KEGG analysis of the 1000 SCLC-related genes. **(C)** Venn diagram predicts potential targets for CE to exert anti-SCLC effects. The PPI network **(D)** of the 60 intersecting targets and subnetwork clusters **(E)** was identified. **(F, G)** GSEA related HALLMARKS **(F)** and Reactome **(G)** analysis of the 60 intersecting targets. **(H)** The hub gene of the PPI network identified by Cytoscape cytoHubba plugin. The degree of gene interactions in the PPI network was calculated by cytoHubba, and colored from yellow to red, indicating the degree from low to high.

To explore the underlying mechanism of CE’s anti-SCLC effects, network pharmacology analysis was utilized. Initially, 1,000 SCLC-related genes were identified from public databases, including GEO, CTD, and GeneCards (Figure 3A; Supplementary Table S5). KEGG pathway enrichment analysis revealed that these genes were primarily enriched in pathways associated with DNA replication, the cell cycle, and mismatch repair. Notably, these genes were also significantly enriched in the small cell lung cancer gene set (Figure 3B). Next, 674 potential target genes of CE were identified and intersected with the 1,000 SCLC-related genes, yielding 60 potential target genes of CE against SCLC (Figure 3C; Supplementary Tables S6, S7). A protein-protein interaction (PPI) network was constructed using the 60 identified genes. After eliminating unconnected nodes, the PPI network included 56 nodes and 166 edges, revealing intricate interconnections among the proteins (Figure 3D). Cluster identification analysis indicated that these genes were primarily involved in G2/M transition of the mitotic cell cycle, cholesterol biosynthesis, VEGFR2-mediated cell proliferation, the renin-angiotensin system, and serotonin clearance from the synaptic cleft (Figure 3E). Furthermore, GSEA Hallmark and Reactome analyses of these genes revealed significant enrichment in cholesterol-related pathways (Figures 3F,G; Supplementary Tables S8, S9). We further examined the core genes identified from the PPI network using the Cytoscape cytoHubba plugin. Interestingly, genes related to cholesterol metabolism, such as SQLE, FDFT1, and IDI1, did not rank highly among the central nodes in the network (Figure 3H). These results suggest that CE may influence SCLC cells through various pathways, particularly by modulating cholesterol metabolism. However, this hypothesis requires further validation, prompting the use of additional techniques to analyze the effects of CE on SCLC cells.

### 3.4 CE disrupts cholesterol metabolism

**FIGURE 4:**
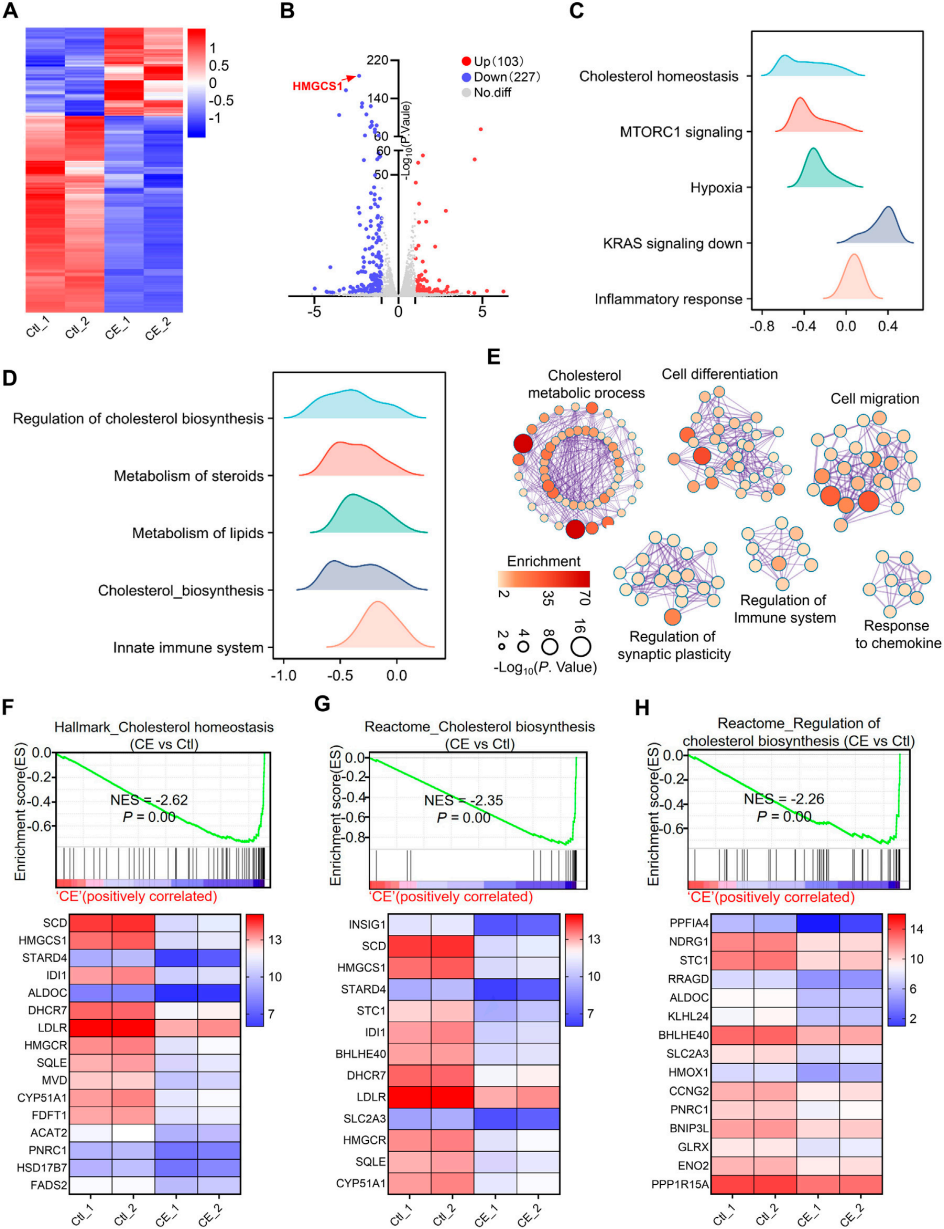
CE disrupts cholesterol metabolism. **(A)** Heatmap shows the expression of genes in RNA-seq data of H1688 cells treated with or without CE (1 µM). **(B)** Volcano plot shows the DEGs identified from RNA-seq of H1688 cells treated with or without CE (1 µM). **(C, D)** Results of the GSEA Hallmark **(C)** and Reactome **(D)** enrichment of pathways after CE exposure are shown. The *X*-axis coordinates represent the enrichment scores. **(E)** Pathway enrichment of the DEGs in H1688 cells treated with or without CE (1 µM). Metascape was used to conduct pathway enrichment; the network was visualized using Cytoscape. Network of enriched terms, circles are Gene Ontology (GO) terms, colored by P value, where terms containing more genes tend to have a more significant P value. **(F, G, H)** Individual GSEA plots and gene expression changes within the corresponding gene sets: Hallmark_Cholesterol Homeostasis **(F)**, Reactome_Cholesterol Biosynthesis **(G)**, and Reactome_Regulation of Cholesterol Biosynthesis **(H)** for H1688 cells treated with or without CE. NES, normalized enrichment score.

To gain deeper insight into the molecular mechanism underlying CE’s anti-SCLC effects, RNA sequencing (RNA-seq) was performed on H1688 cells treated with or without CE (1 µM). This analysis identified 330 differentially expressed genes (DEGs), comprising 227 downregulated and 103 upregulated genes. Among the downregulated genes, HMGCS1, a key rate-limiting enzyme in the cholesterol biosynthesis pathway, was the most significantly affected (Figures 4A,B; Supplementary Table S10). Subsequently, GSEA was utilized to explore the signaling pathways associated with these DEGs. Hallmark enrichment analysis revealed that cholesterol homeostasis, MTORC signaling, and hypoxia gene sets were inhibited following CE treatment, while gene sets related to KRAS signaling and inflammatory responses were activated in response to CE (Figure 4C; Supplementary Table S11). Reactome enrichment analysis further indicated that cholesterol synthesis and the innate immune system were also inhibited post-CE treatment (Figure 4D; Supplementary Table S12). Cytoscape Enrichment Map analysis categorized these DEGs into six main functional groups: cholesterol metabolic process, cell differentiation, cell migration, regulation of synaptic plasticity, regulation of the immune system, and response to chemokines (Figure 4E). Analysis of gene involved in cholesterol metabolism revealed significant downregulation of genes such as HMGCS1, IDI1, HMGCR, SQLE, and FDFT1, following CE treatment (Figures 4F–H), supporting the hypothesis that CE inhibits cholesterol biosynthesis in SCLC cells.

### 3.5 CE inhibits key cholesterol synthesis enzymes and lowers cholesterol levels in SCLC cells

**FIGURE 5:**
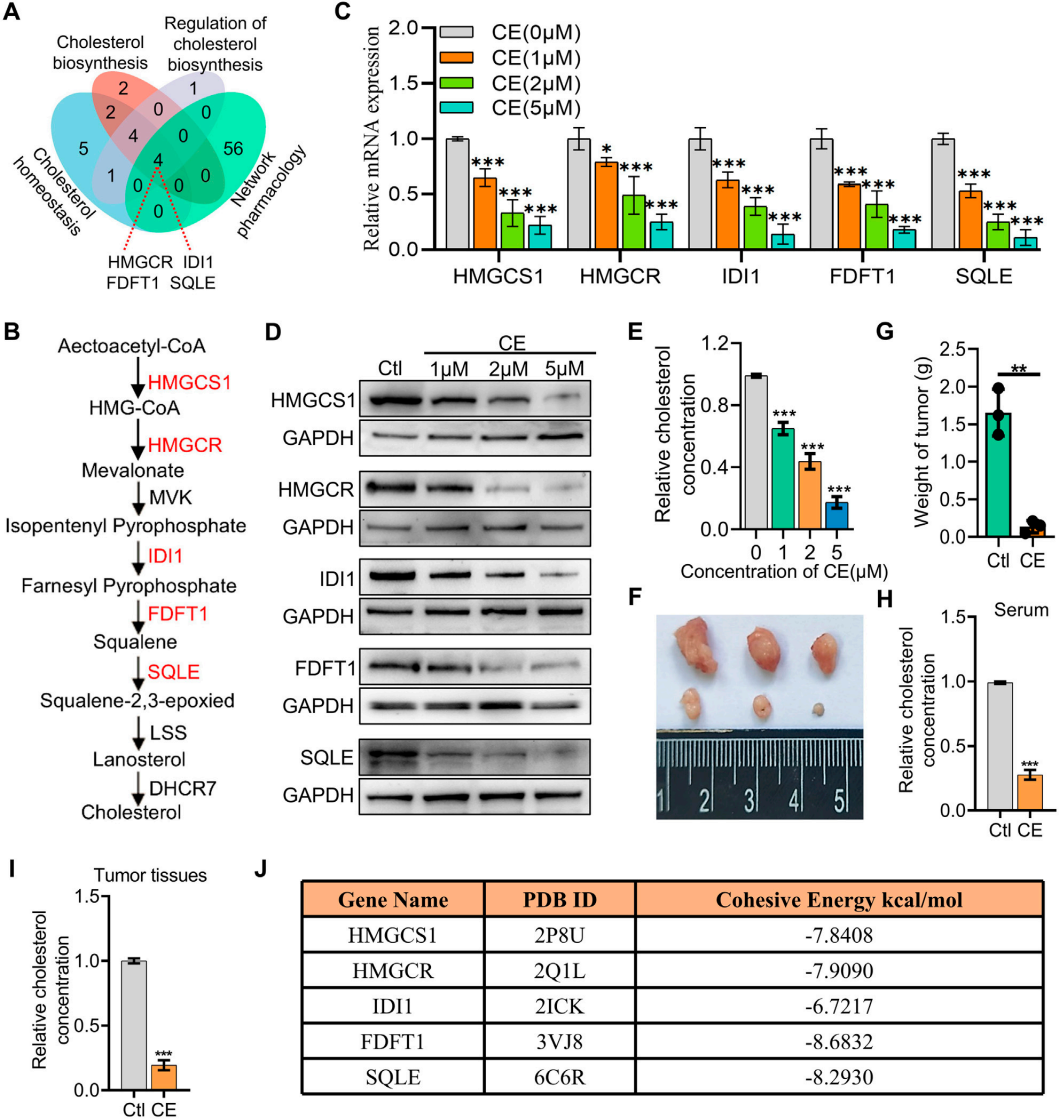
CE inhibits key cholesterol synthesis enzymes and lowers cholesterol levels in SCLC cells. **(A)** Intersection of the DEGs associated with cholesterol metabolism from RNA-Seq data and 60 predicted target genes identified through network pharmacology. **(B)** Schematic representation of the cholesterol biosynthesis pathway, highlighting critical rate-limiting enzymes. **(C)** RT-PCR analysis demonstrating mRNA levels of HMGCS1, HMGCR, IDI1, FDFT1, and SQLE following CE treatment. **(D)** Western blot analysis showing protein levels of the five genes after CE treatment. **(E)** Cholesterol content in H1688 cells treated with or without CE (1 µM) for 24 h. **(F, G)** Representative tumor image **(F)** and tumor weight **(G)** of H1688 cells-derived xenografts treated with or without CE (10 mg/kg), n = 3. **(H, I)** Measurement of cholesterol concentration in serum **(H)** and tumor tissues **(I)** of H1688 cells-derived xenografts. **(J)** The binding energy between corresponding gene and CE. Data are presented as means ± SD of three simultaneously performed experiments **(C, E, H, I)**. *P < 0.05, **P < 0.01, ***P < 0.001.

Both network pharmacology and RNA-Seq data analyses indicate that CE regulates cholesterol metabolism in SCLC cells. To better understand the mechanisms underlying this regulation, the DEGs associated with cholesterol metabolism from the RNA-Seq data were intersected with the 60 predicted target genes identified through network pharmacology, resulting in the identification of four potential key targets: HMGCR, IDI1, FDFT1, and SQLE (Figure 5A). These genes, which are rate-limiting enzymes in the cholesterol biosynthesis pathway (Figure 5B), were prioritized for further analysis. Interestingly, HMGCS1, another key rate-limiting enzyme (Figure 5B), was not predicted by network pharmacology, but it was found to be the most significantly downregulated in the RNA-Seq data. Given the significant downregulation of HMGCS1 in the RNA-Seq data and its crucial role in cholesterol metabolism, HMGCS1 was included in the subsequent analyses, along with the four predicted genes. This combined approach allowed for a comprehensive examination of CE’s impact on cholesterol metabolism in SCLC cells. RT-PCR and Western blot analyses further confirmed that the mRNA and protein levels of all five genes (HMGCS1, HMGCR, IDI1, FDFT1, and SQLE) were significantly reduced following CE treatment (Figures 5C,D). Additionally, the cholesterol content in H1688 cells was also notably decreased after CE treatment (Figure 5E). To validate the *in vivo* anti-SCLC effects of CE, xenograft mouse models were established using H1688 cells. The results demonstrated a significant reduction in tumor volumes (Figure 5F) and tumor weight in the CE-treated group compared to the control group (Figure 5G). Furthermore, cholesterol levels in both serum (Figure 5H) and tumor tissues (Figure 5I) of the xenograft mice were lower after CE treatment, reinforcing the link between CE treatment and reduced cholesterol synthesis. Molecular docking further verified that CE binds strongly to key enzymes in the cholesterol biosynthesis pathway (Figure 5J). These findings suggest that CE’s anti-SCLC effects are mediated by inhibition of cholesterol biosynthesis.

### 3.6 High expression of key cholesterol synthesis enzymes promotes SCLC cell proliferation and correlates with poor prognosis

**FIGURE 6:**
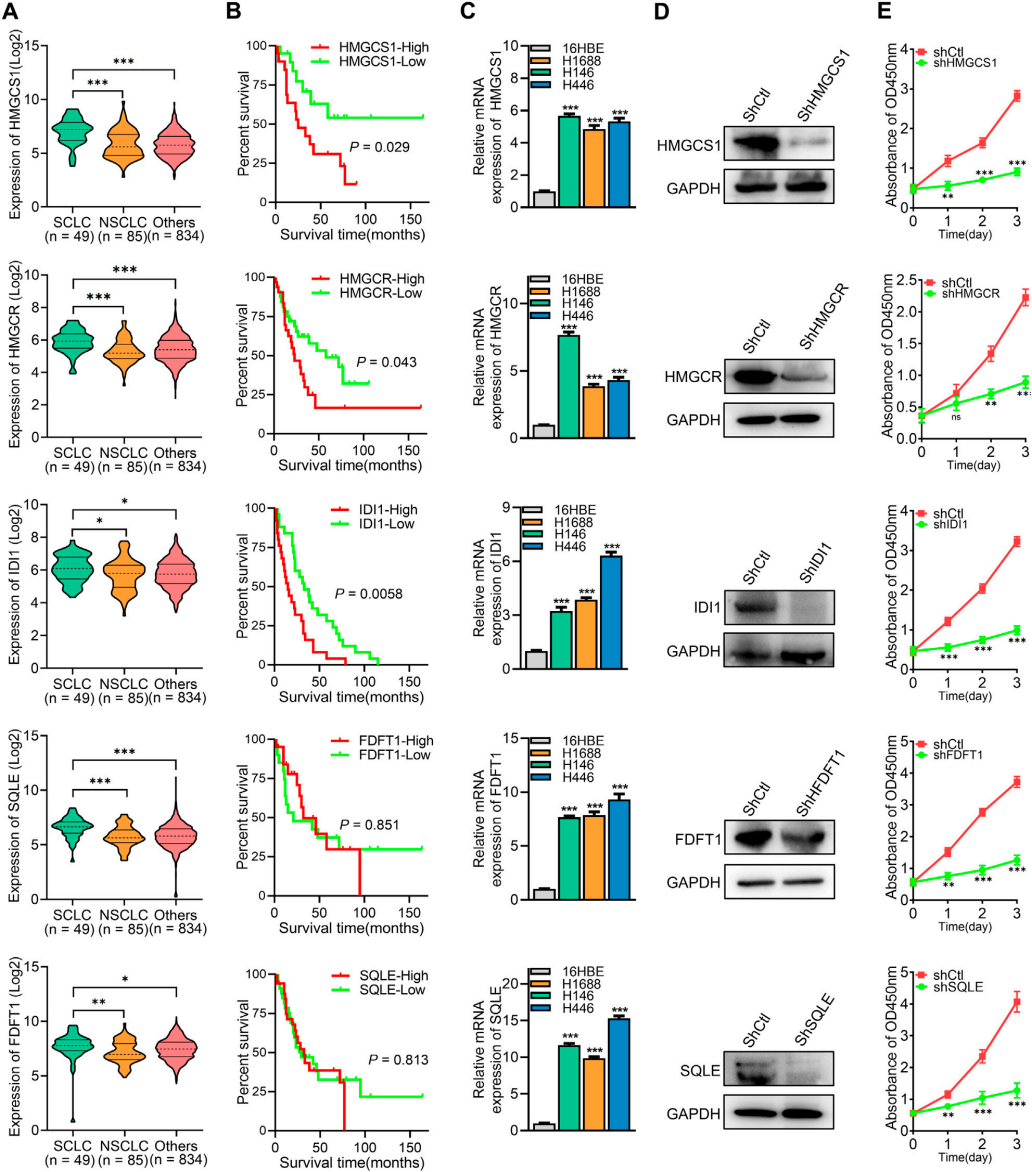
High expression of key cholesterol synthesis enzymes promotes SCLC cell proliferation and correlates with poor prognosis. **(A)** The expression levels of corresponding gene’s mRNA in SCLC, NSCLC, and other cancer cell lines were obtained from the Cancer Cell Line Encyclopedia (CCLE). **(B)** Overall survival analysis of human SCLC samples based on the corresponding gene expression. **(C)** RT-PCR was used to analyze the expression levels of key cholesterol synthesis enzymes (HMGCS1, HMGCR, IDI1, FDFT1, and SQLE) in 16HBE (human bronchial epithelial cell) and SCLC cell lines. **(D)** Protein expression analysis was conducted on the corresponding genes in H1688 cells transfected with either shCtl or shRNA targeting each specific gene. **(E)** Cell proliferation was measured after silencing of HMGCS1, HMGCR, IDI1, FDFT1, and SQLE in H1688 cells. Data are presented as means ± SD of three simultaneously performed experiments **(C, E)**. *P < 0.05, **P < 0.01, ***P < 0.001.

The roles of the five key rate-limiting enzymes (HMGCS1, HMGCR, IDI1, FDFT1, and SQLE) in the cholesterol synthesis pathway in SCLC were further investigated. Analysis of data from the Cancer Cell Line Encyclopedia (CCLE) revealed that the expression levels of HMGCS1, HMGCR, IDI1, FDFT1, and SQLE were significantly higher in SCLC cell lines compared to non-small cell lung cancer (NSCLC) cell lines and other cancer cell lines (Figure 6A). Survival analysis demonstrated that high expression of HMGCS1, HMGCR, and IDI1 was associated with poor prognosis in SCLC patients, while the expression of FDFT1 and SQLE did not significantly impact patient outcomes (*P* > 0.05) (Figure 6B). To confirm these findings, we assessed mRNA expression levels of these five genes in SCLC cells, and the result was consistent with the CCLE data, showing elevated levels of these genes (Figure 6C). To further explore the effects of these genes on SCLC cell proliferation, shRNA was utilized to knock down the expression of each gene in H1688 cells (Figure 6D). Cell viability assays demonstrated that silencing any of these genes significantly inhibited the proliferation of H1688 cells (Figure 6E). In summary, these findings indicate that high expression of key cholesterol synthesis enzymes is a hallmark of SCLC and contributes to the proliferation of SCLC cells.

## 4 Discussion

Small cell lung cancer (SCLC) is characterized by its aggressive nature and poor prognosis, often associated with limited treatment options (Wang et al., 2023). Identifying new therapeutic agents is critical to improving outcomes for SCLC patients. In this study, Cepharanthine (CE) was identified as a promising anti-SCLC agent from a natural compounds library. CE significantly inhibited SCLC cell growth both *in vitro* and *in vivo*. In addition, CE promoted the apoptosis of SCLC cells and inhibited their migration and invasion. To investigate the specific molecular mechanisms underlying CE’s anti-SCLC effects, we employed network pharmacology and RNA-Seq methodologies.

Network pharmacology is a powerful tool in drug discovery, providing insights into drug-biological system interactions (Zhao et al., 2023). It integrates biological data to identify therapeutic targets and pathways, supporting a systems biology approach to drug discovery (Nogales et al., 2022). However, there are several limitations to this approach. Network pharmacology depends on existing databases, which may contain incomplete or inaccurate data, affecting results (Zhang et al., 2023). Additionally, simplifying complex biological systems into static models may also miss the dynamic nature of tumor biology and signaling interactions (Zhao et al., 2023). Furthermore, biases in target selection and challenges in predicting molecular interactions complicate the interpretation of findings (Zhang et al., 2023). In our study, we aimed to explore the mechanisms underlying CE’s anti-SCLC effects using network pharmacology methods. Network pharmacology analysis identified 60 potential target genes related to CE’s anti-SCLC effects. Enrichment analysis indicated that these genes play a significant role in regulating cholesterol metabolism. Although significant, cholesterol-related genes were not among the top hub genes, highlighting a limitation in traditional network pharmacology methods. The lower ranking of these genes may result from the complexity of their regulatory networks and interactions with other pathways, which static models cannot fully capture. This emphasizes the need for complementary methods to better elucidate the mechanisms by which CE exerts its effects.

RNA-seq is a powerful tool for profiling gene expression changes and discovering novel targets and pathways in disease (Wang et al., 2009). Its ability to capture both known and unknown transcripts, quantify gene expression with high precision, and provide insights into transcript structure makes it particularly valuable for complex diseases like cancer (Stark et al., 2019). However, the large volume of data generated can complicate the identification of the most relevant biological insights, particularly when distinguishing true signals from background noise (Mcdermaid et al., 2019). In this study, we combined RNA-seq and network pharmacology to explore the mechanisms underlying CE’s anti-SCLC activity. This integrative approach allowed us to overcome the limitations of both methods: RNA-seq offered a comprehensive view of potential gene expression changes, while network pharmacology helped prioritize key targets and pathways. Our analysis integrating network pharmacology and RNA-seq data suggests that CE inhibits cholesterol synthesis in SCLC cells by downregulating key enzymes in the cholesterol biosynthesis pathway, including HMGCR, IDI1, FDFT1, and SQLE. Despite not being predicted by network pharmacology, HMGCS1, a key enzyme in the pathway, was included in our analyses due to the reliability of the RNA-seq data. This highlights the advantage of RNA-seq in uncovering biologically relevant genes that may not be prioritized in network pharmacology. Importantly, our results show that even genes not ranked as top hubs in network pharmacology can have crucial roles in therapeutic responses. This finding emphasizes the complementary nature of network pharmacology and RNA-seq, offering a more robust framework for identifying potential therapeutic targets.

Cholesterol metabolism is crucial in tumor progression, influencing key aspects of cancer biology such as cell proliferation, migration, and evasion of apoptosis (Xu et al., 2020; Huang et al., 2020). Moreover, cholesterol metabolism plays a key role in SCLC biology. For instance, low serum low-density lipoprotein (LDL) levels and reduced LDL receptor (LDLR) expression are associated with better overall survival in SCLC patients (Zhou et al., 2017). Cholesterol is also crucial for signaling pathways, like the Hedgehog pathway, that drive SCLC progression (Chen et al., 2018). Depletion of cholesterol may disrupt these pathways and impair cell survival, particularly through changes in protein prenylation, which is vital for RAS signaling and other pathways (Ahmadi et al., 2017; Kashiwagi et al., 2022). Cholesterol synthesis is a complex process mainly occurring in the liver and intestines. It begins with the conversion of acetyl-CoA to mevalonate, catalyzed by two key enzymes: HMG-CoA synthase 1 (HMGCS1) and HMG-CoA reductase (HMGCR). Mevalonate is then transformed into farnesyl pyrophosphate through the action of isopentenyl-diphosphate delta-isomerase 1 (IDI1). Subsequent reactions, involving enzymes such as farnesyl-diphosphate farnesyltransferase 1 (FDFT1) and squalene epoxidase (SQLE), lead to the final production of cholesterol (Liu et al., 2023b; Luo et al., 2020). These enzymes play a crucial role in cholesterol biosynthesis and are potential therapeutic targets in tumor progression (Lu et al., 2023a). In our study, we found that CE significantly inhibits the expression of key rate-limiting enzymes (HMGCS1, HMGCR, IDI1, FDFT1, and SQLE), thereby reducing cholesterol synthesis in SCLC cells. This finding supports previous research showing that overexpression of these enzymes is linked to enhanced tumor growth and poor prognosis in various cancers. Validation using the public CCLE database confirmed high expression of these five genes in SCLC cells. Notably, high levels of HMGCS1, HMGCR, and IDI1 were associated with worse prognosis. Furthermore, loss-of-function experiments revealed that silencing these genes significantly inhibited SCLC cell proliferation, highlighting their role as oncogenes.

In addition to its role in cholesterol metabolism, our network pharmacology analysis identified several pathways that may contribute to the anti-SCLC effects of CE, all of which are significant in SCLC development and progression. For instance, the VEGFR2-mediated pathway is crucial for tumor angiogenesis and cell proliferation, promoting tumor growth and metastasis by enhancing blood supply (Simons et al., 2016). The regulation of the G2/M transition in the cell cycle is also critical for cellular proliferation, with dysregulation often observed in cancers (Matthews et al., 2022). Furthermore, pathways related to the renin-angiotensin system may influence the tumor microenvironment, affecting cell signaling and tumor behavior (Catarata et al., 2020). While our network pharmacology findings highlight the importance of these pathways in CE’s anti-SCLC effects, further experimental validation is needed. Investigating CE’s interaction with these pathways will provide further insights into its mechanisms and therapeutic potential.

Our study identifies CE as a potential therapeutic agent against SCLC. It exerts this effect by inhibiting cholesterol synthesis through downregulating key enzymes, including HMGCS1, HMGCR, IDI1, FDFT1, and SQLE. However, several limitations should be addressed in future research. The mechanisms by which cholesterol metabolism contributes to CE’s anti-SCLC effects require further *in vivo* and *in vitro* investigation. Additionally, the bioavailability and metabolic pathways of CE should be better understood to optimize its clinical application. Chemical modifications could also improve CE’s pharmacokinetics, enhancing stability and bioavailability. Future studies should involve larger sample sizes to evaluate CE’s *in vivo* effects, assess dose-dependent outcomes, and compare its efficacy with current chemotherapy agents. Investigating potential synergistic effects of CE with other chemotherapies could further improve treatment strategies for SCLC. Addressing these areas will maximize CE’s therapeutic potential and improve outcomes for patients.

## 5 Conclusion

Our research demonstrates that CE disrupts cholesterol metabolism by downregulating key rate-limiting enzymes involved in cholesterol synthesis: HMGCS1, HMGCR, IDI1, FDFT1, and SQLE. These findings provide a new treatment option for SCLC patients and identify critical therapeutic targets associated with cholesterol metabolism. These findings open new avenues for research targeting these enzymes, which could enhance treatment strategies and improve patient outcomes.

## Data Availability

The datasets presented in this study can be found in online repositories. The names of the repository/repositories and accession number(s) can be found in the article/Supplementary Material.

## Abbreviations

SCLC, Small cell lung cancer; CE, Cepharanthine; KEGG, Kyoto encyclopedia of genes and genomes; DMSO, Dimethyl sulfoxide; GAPDH, Glyceraldehyde-3-phosphate dehydrogenase; IC50, 50% inhibition of cell viability; PPI, Protein-Protein interaction; DEGs, Differentially expressed genes; LogFC, LogFoldChange; GEO, Gene Expression Omnibus; GSEA, Gene Set Enrichment Analysis; CCK-8, Cell Counting Kit-8; qRT‒PCR, Quantitative Rea-time PCR; shRNA, Short hairpin RNA; STRING, Search Tool for the Retrieval of Interacting Genes/Proteins.
